# Infertility revealing intratubular germinal cell neoplasia of the testis: Case report and review of the literature

**DOI:** 10.1016/j.ijscr.2023.108609

**Published:** 2023-08-02

**Authors:** Ahmed Jdaini, Anouar El Moudane, Hammou El Farhaoui, Nadir Miry, Amal Bennani, Ali Barki

**Affiliations:** a Department of Urology, Mohammed IV University Medical Center, Mohammed the First University Oujda, Morocco; b Pathology Department, Mohammed VI university hospital, Oujda, Morocco

**Keywords:** Testis, Germ cell tumor, Intratubular neoplasia, Seminoma, Case report

## Abstract

**Introduction:**

Testicular cancer is the most frequent solid tumor in young people. Most germ cell tumors of the testis, with the exception of vitelline tumors and teratomas in children, and spermatocytic seminoma in the elderly, derive from a common precursor. This precursor, initially described as carcinoma in situ (CIS), is now referred to as intratubular Germinal Cell Neoplasia (ITGCN).

**Case presentation:**

We report the case of a 37-year-old man with intratubular Germinal Cell Neoplasia (ITGCN) on a testis already treated for cryptorchidism in a context of infertility. We proposed active surveillance, but the patient preferred radiotherapy.

**Discussion:**

The origin of ITGCN is still not fully understood. The detection of ITGN is often incidental since it typically does not present with noticeable symptoms, and clinical examination may appear normal. The standard treatment for ITGCN is scrotal radiotherapy.

**Conclusion:**

The standard treatment for ITGCN is scrotal radiotherapy. However, for patients who desire to preserve fertility, regular observation may be considered as an alternative.

## Highlights

• Testicular cancer is the most frequent solid tumor in young people
• ITGN lesions indicate a pre-invasive tumor stage, distinguished by the proliferation of dysplastic cells within the seminiferous tubules, while the basement membrane remains uninvaded
• Imaging techniques, such as ultrasound, have limited utility in directly detecting ITGN
• Radiation therapy, specifically with a dose of 18 to 20 Gy, has been associated with superior eradication of germ cell neoplasia in situ compared to chemotherapy

## 1 Introduction

Intratubular Germinal Cell Neoplasia (ITGCN) is a precancerous lesion that shares similarities with carcinoma in situ regarding epithelial lesions. Germ cell neoplasia is a precursor of testicular cancer. This histology is occasionally seen in the diagnostic biopsy of an infertile man. The prevalence of this condition is 0–1 % in infertile men and is 2–4 % in men with cryptorchidism.

ITGCN is a disorder characterized by the presence of abnormal cells in the small tubules where sperm cells initiate their development. These aberrant cells have the potential to transform into cancerous cells and metastasize to the adjacent healthy tissue. Therefore, it is crucial to recognize and effectively manage this pathological condition, as it can progress to invasive germ cell tumors. In our specific case, we present a patient who was diagnosed in our university center with intratubular Germinal Cell Neoplasia (ITGCN) during an infertility test, which is a therapeutic challenge to preserve the testicle. We proposed active surveillance, but the patient preferred to undergo external radiotherapy. The work has been reported in line with the SCARE 2020 criteria [5].

## 2 Case report

**Fig. 1:**
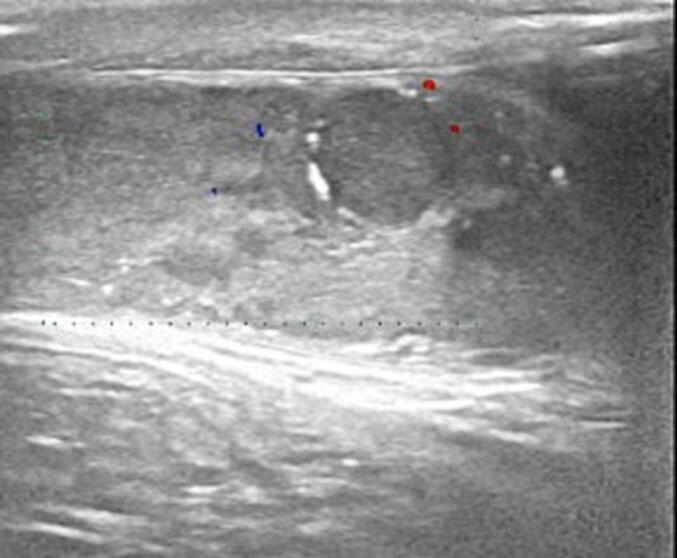
Ultrasound showed heterogenous zones on the right testicle measuring 16 mm.

A 37-year-old man, with a history of surgery for right cryptorchidism at age 8 and no other medical history, consulted our department for primary infertility for 3 years. Clinical examination revealed a hypotrophic right testicle with a left varicocele and minor pain in the left testis, Ultrasound showed a heterogenous zones on the right testicle measuring 16 mm. (Fig. 1), and testicular tumor markers were: alpha fetoprotein (AFP) 5.52 ng/mL (N: 0–13.4), human chorionic gonadotropin (HCG) < 2.3 mIU/mL (N: 0–5), and lactate dehydrogenase (LDH) 215 U/ L (N: 125–243).

Cytological analysis of the sperm showed no abnormalities that could help us make a diagnosis.

**Fig. 2:**
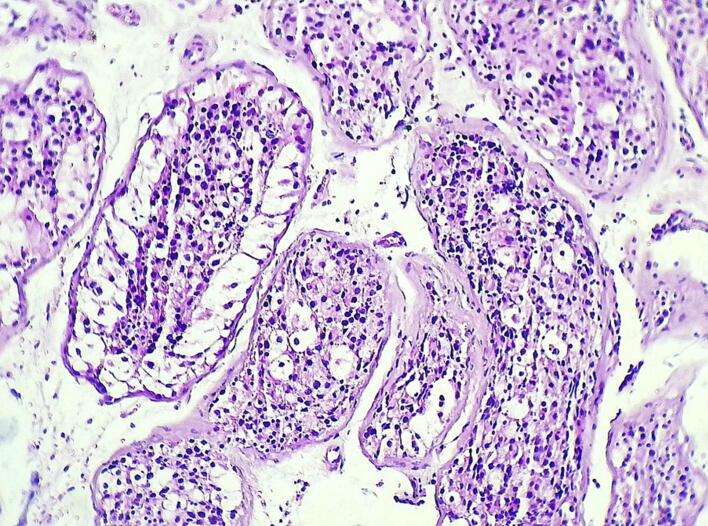
Photomicrograph showing multiple seminiferous tubules containing large atypical cells with abundant eosinophilic cytoplasm; the nuclei are hyperchromatic and angulated with prominent nucleoli (H&E, ×100).

An inguinal right testicular biopsy was performed for anatomopathological study. The photomicrograph showing multiple seminiferous tubules containing large atypical cells with abundant eosinophilic cytoplasm; nuclei are hyperchromatic and angular with prominent nucleoli (H&E, ×100), suggestive of intratubular germinal neoplasia (Fig. 2).

We proposed surveillance for our patient because he has an infertility, but the patient wishes to have radiotherapy for his intratubular germinal neoplasia.

The patient received 20 Gy of irradiation in ten fractionated sessions by oncologists, with disappearance of the intratubular germinal neoplasia on control imaging, the patient was satisfied with the treatment, especially with the preservation of his testicle.

## 3 Discussion

ITGCN lesions indicate a pre-invasive tumor stage, distinguished by the proliferation of dysplastic cells within the seminiferous tubules, while the basement membrane remains uninvaded [1]. Importantly, the morphology of Sertoli and Leydig cells appears to be unaffected and remains normal.

Immunohistochemical analysis reveals that the dysplastic cells continue to express placental alkaline phosphatase (PLAP) and c-kit (CD117) [2].

The origin of ITGCN is still not fully understood, and the available information on its precise etiology remains incomplete. However, it is generally accepted that the abnormal cells associated with ITGCN are believed to arise at an early stage, potentially during the antenatal period. Further research is needed to gain a comprehensive understanding of the exact mechanisms underlying the development of ITGCN. Risk factors for developing ITGCN are the existence of a contralateral tumor, history of cryptorchidism, infertility as described in our case, gonadic dysgenesis (4 6 XX/4 6 XY, 45X/46XY and female phenotype), and the majority of cases in the literature concern patients aged between 20 and 40.

The detection of ITGCN is often incidental since it typically does not present with noticeable symptoms, and clinical examination may appear normal, except for a hypotrophic (underdeveloped) testicle. In such cases, a biopsy is the only reliable option for confirming the diagnosis. This procedure involves obtaining a tissue sample from the affected area for microscopic examination, allowing for an accurate identification of ITGCN. In post-pubertal individuals, a biopsy with a diameter of 3 mm, which samples 0.1 % of the testicular volume, can provide a 100 % accurate diagnosis if >10 % of the seminiferous tubules are involved in the ITGCN process [3].

Imaging techniques, such as ultrasound, have limited utility in directly detecting ITGCN. However, ultrasound may sometimes reveal heterogeneous zones within the testicles that correspond to areas affected by ITGCN. These findings can help identify potential candidates for further investigation and biopsy. While ultrasound alone cannot definitively diagnose ITGCN, it can assist in targeting the suspicious areas for a more accurate and focused biopsy.

ITGCN can undergo progression and transform into either seminoma or non-seminomatous germ cell tumors, with the exception of spermatocytic seminoma. This process of dedifferentiation can occur either before or after crossing the basement membrane,

Treatment of ITGCN is crucial when incidentally discovered in testis biopsy due to its classification as a premalignant lesion. The risk of ITGCN progressing to malignant germ cell tumors is approximately 50 % within 5 years and 70 % within 7 years.

There are several treatment options for ITGCN including radiotherapy, chemotherapy, orchiectomy or intensive surveillance.

Radiation therapy, specifically with a dose of 18 to 20 Gy, has been associated with superior eradication of germ cell neoplasia in situ compared to chemotherapy [4].

Chemotherapy has shown potential in eradicating germ cell neoplasia in situ in a significant number of patients who receive it as adjuvant treatment for primary germ cell tumors. Studies have reported that up to two-thirds of patients undergoing chemotherapy achieve eradication of germ cell neoplasia in situ [4]. However, carboplatin-based treatment regimens have shown positive disease on repeat biopsies in 66 % to 75 % of cases, and treatment-related hypogonadism rates range from 30.8 % to 38.5 % [4].

The desire to preserve fertility makes it rational to adopt a simple monitoring. However this surveillance must be close and prolonged, because evolution to infiltrative tumors is observed in >50 % of cases. This management is acceptable only in compliant patients, who must be informed of the risk of anorchidism if a tumor develops during follow-up. Monitoring is clinical and ultrasonographic. The discovery of any abnormality requires histological examination.

It is important to note that chemotherapy is generally less effective compared to radiotherapy. The management approach for ITGCN depends on factors such as the patient's age, the presence of risk factors, and the existence of a primary germ cell tumor, also include information pertaining to the recovery of spermatogenesis following radiation therapy. E.g. it hinges on several factors, including dosage, use of adjuvant chemotherapy, pretreatment fertility potential.

## 4 Conclusion

ITGCN has been recognized as a precursor lesion in the development of germinal tumors, providing valuable insights into their pathogenesis. In such cases, a bifocal surgical biopsy is indicated.

The standard treatment for ITGCN is scrotal radiotherapy. However, for patients who desire to preserve fertility, regular observation may be considered as an alternative. Orchiectomy is a topic of discussion in specific cases, particularly those involving sexual ambiguity with an XY genotype.

## Ethical approval

The ethical approval has been exempted by our institution.

The patient gave written permission to publish his case findings.

## Funding

No.

## CRediT authorship contribution statement

Ahmed Jdaini, Anouar El moudane, Hammou El farhaoui: write the paper.

Nadir Miry, Amal Bennani were involved in the histopathlogical analysis.

Ali Barki supervised the paper writing.

## Guarantor

N/A.

## Research registration (for case reports detailing a new surgical technique or new equipment/technology)

N/A.

## Consent

Written informed consent was obtained from the patient for publication of this case report and accompanying images. A copy of the written consent is available for review by the Editor-in-Chief of this journal on request.

## Provenance and peer review

Not commissioned, externally peer-reviewed.

## Declaration of competing interest

The authors declare that there is no conflict of interests regarding the publication of this articale

## Data Availability

Available.
